# Machine Learning and Mendelian Randomization Reveal Molecular Mechanisms and Causal Relationships of Immune-Related Biomarkers in Periodontitis

**DOI:** 10.1155/mi/9983323

**Published:** 2024-12-16

**Authors:** Yuan Li, Bolun Zhang, Dengke Li, Yu Zhang, Yang Xue, Kaijin Hu

**Affiliations:** ^1^State Key Laboratory of Oral and Maxillofacial Reconstruction and Regeneration, National Clinical Research Center for Oral Diseases, Shaanxi Clinical Research Center for Oral Diseases, Department of Oral and Maxillofacial Surgery, School of Stomatology, The Fourth Military Medical University, Xi'an, China; ^2^Department of Stomatology, School of Stomatology, The Third Affiliated Hospital, Xi'an Medical University, Xi'an, China

**Keywords:** biomarkers, machine learning, Mendelian randomization, periodontitis, single-cell sequencing

## Abstract

This study aimed to investigate the molecular mechanisms of periodontitis and identify key immune-related biomarkers using machine learning and Mendelian randomization (MR). Differentially expressed gene (DEG) analysis was performed on periodontitis datasets GSE16134 and GSE10334 from the Gene Expression Omnibus (GEO) database, followed by weighted gene co-expression network analysis (WGCNA) to identify relevant gene modules. Various machine learning algorithms were utilized to construct predictive models, highlighting core genes, while MR assessed the causal relationships between these genes and periodontitis. Additionally, immune infiltration analysis and single-cell sequencing were employed to explore the roles of key genes in immunity and their expression across different cell types. The integration of machine learning, MR, and single-cell sequencing represents a novel approach that significantly enhances our understanding of the immune dynamics and gene interactions in periodontitis. The study identified 682 significant DEGs, with WGCNA revealing seven gene modules associated with periodontitis and 471 core candidate genes. Among the 113 machine learning algorithms tested, XGBoost was the most effective in identifying periodontitis samples, leading to the selection of 19 core genes. MR confirmed significant causal relationships between CD93, CD69, and CXCL6 and periodontitis. Further analysis showed that these genes were correlated with various immune cells and exhibited specific expression patterns in periodontitis tissues. The findings suggest that CD93, CD69, and CXCL6 are closely related to the progression of periodontitis, with MR confirming their causal links to the disease. These genes have potential applications in the diagnosis and treatment of periodontitis, offering new insights into the disease's molecular mechanisms and providing valuable resources for precision medicine approaches in periodontitis management. Limitations of this study include the demographic and sample size constraints of the datasets, which may impact the generalizability of the findings. Future research is needed to validate these biomarkers in larger, diverse cohorts and to investigate their functional roles in the pathogenesis of periodontitis.

## 1. Introduction

Periodontitis is a prevalent chronic inflammatory disease that primarily affects the soft tissues and bone structures surrounding the teeth, impacting approximately 45%–50% of adults worldwide [1, 2]. It is not only the sixth most common disease globally, but severe periodontitis also affects about 11.2% of the population [3]. The onset of periodontitis is primarily driven by a complex interaction between oral bacterial infection and the host immune response, and it is further influenced by individual behavioral habits and overall health status [4, 5]. In addition to causing serious oral health issues, periodontitis is closely associated with systemic health problems such as cardiovascular disease, diabetes, and preterm birth, posing a significant public health challenge [6–9].

Traditional studies on periodontitis typically rely on clinical and pathological indicators to diagnose and assess the severity of the disease [10, 11]; however, these methods often fall short in revealing the underlying molecular mechanisms of the disease. In recent years, the application of machine learning technology has brought significant advances to periodontitis research [12–14]. By analyzing large-scale biomedical data, particularly in the field of bioinformatics, machine learning has the ability to identify potential disease biomarkers and pathological processes, thereby, enhancing the accuracy of disease prediction and facilitating the development of personalized treatments [15–18].

Mendelian randomization (MR) is a powerful tool used to infer causal relationships between exposure factors and disease outcomes [19–22]. MR methods have been widely used to identify molecular markers that contribute to disease development, meaning that gene expression quantitative trait loci (eQTL) variants can be used as instrumental variables to infer causal relationships between genes and diseases [23–25]. Although previous studies have focused on transcriptomic data analysis of periodontitis [26–28], they often overlook the exploration of potential causal relationships between genes and periodontitis. In this study, we integrated transcriptomic and genomic data to comprehensively investigate the molecular mechanisms of periodontitis using machine learning and weighted gene co-expression network analysis (WGCNA) [29]. We employed single-cell RNA sequencing to reveal the expression patterns of key genes in different immune cells and used MR to infer causal relationships between these genes and periodontitis. Our study addresses this gap by employing both MR and advanced machine learning techniques to rigorously analyze periodontitis-associated datasets. This integration allows us to more accurately infer causal relationships and identify key immune-related biomarkers for periodontitis. Unlike much prior research, we apply these methodologies comprehensively, combining them with targeted single-cell RNA sequencing to elucidate the expression patterns of these biomarkers in various immune cells. This approach enhances our understanding of the roles these genes play in the pathogenesis of periodontitis, providing a robust basis for developing more effective diagnostic and therapeutic strategies. Overall, by integrating multi-omics data, we deeply explored immune-related feature genes associated with periodontitis, identifying new biomarkers that provide valuable resources for the precise diagnosis and treatment of periodontitis.

## 2. Materials and Methods

### 2.1. Data Acquisition and Preprocessing

**Figure 1:**
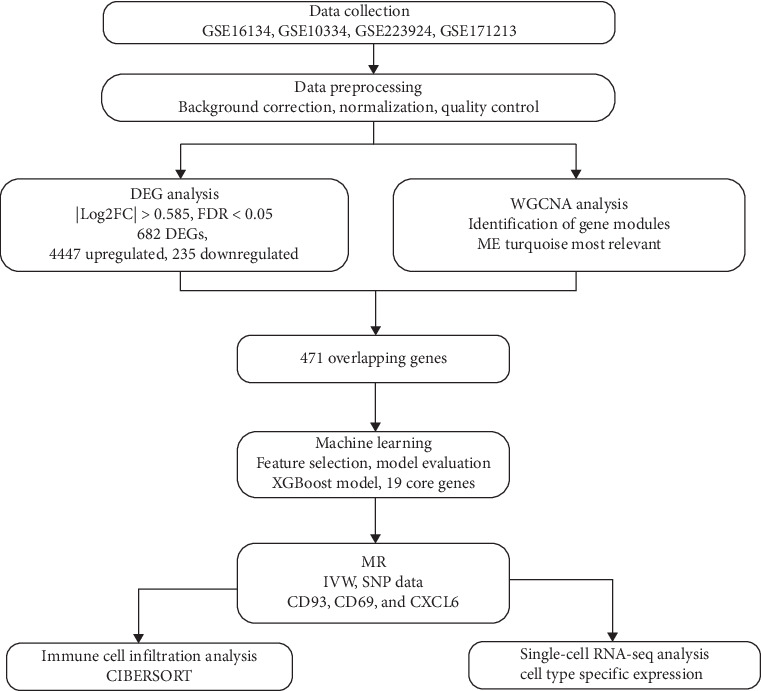
Workflow of this study. MR, Mendelian randomization; WGCNA, weighted gene co-expression network analysis.

This study utilized three periodontitis-related transcriptome datasets from the Gene Expression Omnibus (GEO) database: GSE16134, GSE10334, and GSE223924, as well as single-cell RNA sequencing data from five periodontitis cases in the GSE171213 dataset. GSE16134 includes 310 samples from 120 participants who provided a minimum of two interproximal gingival papillae from maxillary posterior sites, exploring the association between subgingival bacterial profiles and gingival tissue gene expression. GSE10334 contains 247 samples from 90 participants, examining gene expression in both healthy and diseased gingival tissues, providing insights into the transcriptomic changes associated with periodontal health and disease. GSE223924 comprises 20 samples from 20 participants, enhancing the diversity of the periodontal conditions studied. Data preprocessing steps included background correction, quality control, and batch effect elimination using the “sva” package in R to ensure the reliability of the analysis data [30]. The study design is illustrated in Figure 1.

### 2.2. Differentially Expressed Genes (DEGs) Analysis

The limma package was used to analyze DEGs between periodontitis samples and healthy controls [31]. We set |Log2FC| > 0.585 and false discovery rate (FDR) <0.05 as the significance thresholds to filter statistically significant DEGs. The results were visualized using heatmaps and volcano plots for clearer presentation.

### 2.3. WGCNA

To identify gene modules associated with periodontitis, we constructed a co-expression network using the WGCNA package [32]. A soft-thresholding power of five was selected to ensure the scale-free topology of the network. Correlation analysis was then used to identify gene modules most associated with the periodontitis phenotype, followed by further analysis of key genes within these modules. Accurate parameter settings were applied to ensure the identification of functionally relevant gene modules.

### 2.4. GO and KEGG Pathway Enrichment Analysis

To reveal the primary roles of significantly DEGs in biological processes, cellular components, and molecular functions, GO and KEGG enrichment analyses were performed using the clusterProfiler package [33]. Additionally, KEGG pathway analysis was conducted to explore the major biochemical pathways potentially involved in the pathogenesis of periodontitis.

### 2.5. Machine Learning Analysis

To optimize feature selection of key genes and improve the accuracy of periodontitis classification, a comprehensive machine learning framework was adopted, integrating 113 different algorithm combinations. These algorithms included, but were not limited to, elastic net, lasso regression, ridge regression, stepwise generalized linear model (Stepglm), random forest (RF), gradient boosting machine (GBM), support vector machine (SVM), decision tree (DT), naive bayes (NB), K-nearest neighbors (KNN), and XGBoost, covering a range of both classical and modern machine learning models. We applied these algorithm combinations to input features derived from DEGs identified through limma analysis and key module genes identified through WGCNA.

XGBoost was selected for its superior handling of complex datasets with numerous features and missing values. It outperformed other models in classification accuracy, evidenced by the highest average area under the receiver operating characteristic (ROC) curve (AUC) in tests, and its robustness against overfitting, even with extensive hyperparameter tuning, confirmed its effectiveness for analyzing complex patterns in periodontitis. We utilized the xgboost package in R to implement the XGBoost model. Key hyperparameters were optimized using a grid search method. We experimented with learning rates ranging from 0.01 to 0.3, tree depths from 3 to 10, and boosting rounds from 100 to 1000 to balance model accuracy and overfitting.

During model training and evaluation, leave-one-out cross-validation (LOOCV) was employed to ensure model robustness and generalizability. The final model performance was assessed using ROC curves, with the AUC serving as a metric for classification ability. Throughout the analysis, various algorithm combinations were thoroughly compared, and the model with the highest average AUC was selected as the optimal model for feature selection and classification prediction of periodontitis-related key genes.

### 2.6. MR Analysis

MR analysis was conducted using the TwoSampleMR package [34]. Single nucleotide polymorphism (SNP) data were obtained from publicly available eQTL and protein QTL (pQTL) databases, selecting SNPs with genome-wide significance (*p*  < 5 × 10-8) as instrumental variables, while excluding those significantly associated with other potential confounders. To ensure the independence of instrumental variables, linkage disequilibrium (LD) pruning (*R*^2^ < 0.01, window size of 10,000 kb) was employed to exclude SNPs in significant LD. Exposure data were sourced from the eQTL data in the publicly accessible IEU Open GWAS database (https://gwas.mrcieu.ac.uk/) and the pQTL data from deCODE Genetics (https://www.decode.com/summarydata/), while outcome data were obtained from the FinnGen database (https://www.finngen.fi/en/access_results) containing periodontitis GWAS summary data [29, 35]. The significance threshold for the analysis results was set at *p*  < 0.05 using the IVW method. Additionally, Cochran's *Q* test was used to assess heterogeneity, helping to understand whether the instrumental variables used are valid. A *p*-value of less than 0.05 in heterogeneity tests may indicate potential pleiotropy or invalid instruments. Sensitivity analysis was performed using leave-one-out analysis to ensure the robustness of causal inference. The MR-Egger intercept was also employed to test for directional pleiotropy. An intercept significantly different from zero (*p*-value < 0.05) suggests the presence of pleiotropy. This comprehensive approach allowed us to integrate and validate the causal relationships between identified biomarkers and periodontitis, reinforcing the findings from our machine learning and single-cell analyses.

### 2.7. Immune Cell Infiltration Analysis

Immune cell infiltration in periodontitis patients and healthy controls was analyzed using the CIBERSORT method [36]. We used normalized gene expression data to minimize technical differences between samples. We performed a CIBERSORT analysis with 1000 permutations to robustly estimate the proportions of various immune cell types, applying a strict *p*-value threshold of less than 0.05 to ensure that only significant differences in cellular composition were considered. Furthermore, the correlations between the key inflammatory genes CD93, CD69, and CXCL6 and various immune cell types were explored. This analysis aimed to uncover the potential mechanisms by which these genes regulate immune responses in periodontitis.

### 2.8. Single-Cell RNA Sequencing Data Analysis

Single-cell RNA sequencing data from the GSE171213 dataset were processed using the Seurat package [37], including quality control, normalization, identification of variable features, dimensionality reduction, and cell clustering. We performed quality control to filter cells based on gene expression and mitochondrial content, removing outliers to ensure robust downstream analysis. Normalization was conducted using the LogNormalize method, and 1500 variable features were identified for further analysis. We employed principal component analysis (PCA) to reduce dimensionality, selecting significant principal components for cluster identification and visualization with UMAP and violin plots. These plots were used to analyze the expression differences of CD93, CD69, and CXCL6 genes across different cell types and further investigate their associations with periodontitis.

### 2.9. Gene Set Enrichment Analysis (GSEA)

Based on the expression levels of the characteristic genes, samples were divided into high and low expression groups. The GSEA function in the clusterProfiler package was used to calculate enrichment scores for gene sets across these sample groups, thereby identifying biological pathways significantly enriched between the high and low expression groups [38]. The significance thresholds for GSEA results were set at *p*  < 0.05, |normalized enrichment score (NES)| > 1, and FDR <0.25, with analysis results visualized using the enrichplot package. Gene set variation analysis (GSVA) analysis was conducted using the GSVA package, an unsupervised GSVA method, to quantify the enrichment of gene sets within each sample, thereby, assessing the relative activity of gene sets across different samples [39].

### 2.10. Statistical Methods

All statistical analyses were conducted in the R software environment. Comparisons of continuous variables were performed using the Wilcoxon rank-sum test, while categorical variables were analyzed using the Chi-square test. To address the issue of multiple comparisons, the FDR method was employed to adjust *p*-values, ensuring the statistical significance of the results. ROC curves were used to evaluate the diagnostic performance of the models.

## 3. Results

### 3.1. Identification of Periodontitis-Related Genes

The flowchart provides a clear overview of the entire analysis process, showing the main steps and results from data acquisition to final interpretation. It includes an explanation of how different datasets and computational methods are integrated (Figure 1).

**Figure 2:**
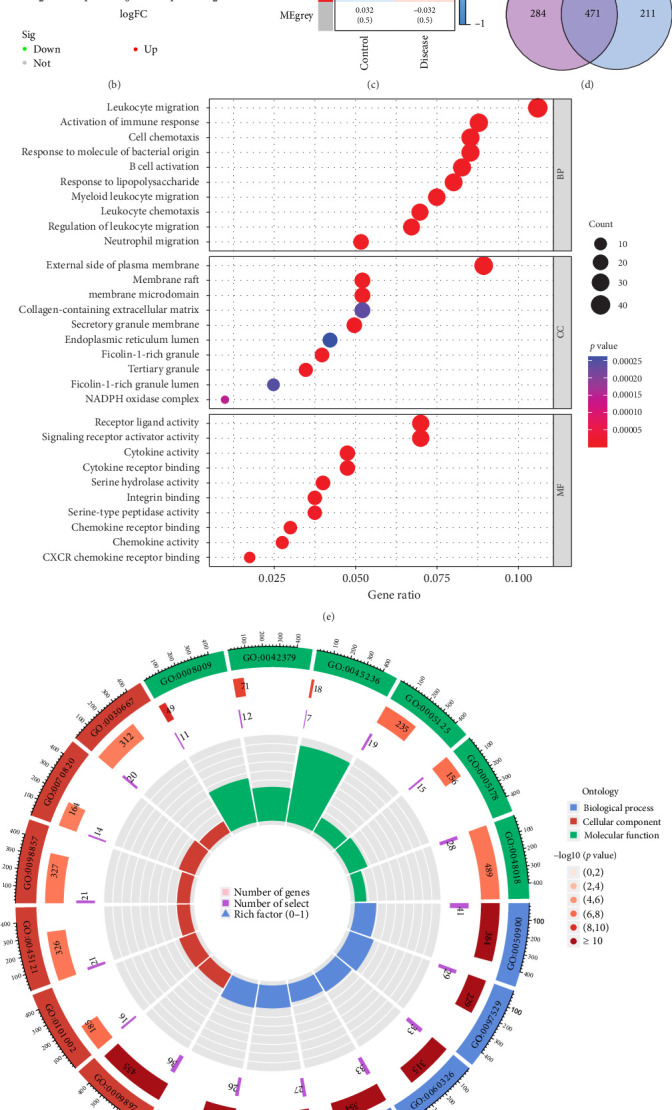
Screening of periodontitis-related genes. (A) Heatmap of differential expression analysis for GSE16134 and GSE10334. (B) Volcano plot of differential expression analysis for GSE16134 and GSE10334. Green represents downregulated genes and red represents upregulated genes. (C) Module–trait heatmap showing the correlation between clustered gene modules and periodontitis, with corresponding correlation coefficients and *p*-values for each module. (D) Venn diagram revealing 471 overlapping candidate hub genes. (E, F) GO enrichment analysis of the candidate hub genes. (G) KEGG pathway analysis of the candidate hub genes. WGCNA, weighted gene co-expression network analysis.

This study utilized the periodontitis datasets GSE16134 (310 samples from 120 participants) and GSE10334 (247 samples from 90 participants) from the GEO database as data sources. After initial screening, we identified 682 significantly DEGs in the periodontitis group compared to the control group, including 447 upregulated and 235 downregulated genes (Figure 2A,B). Through WGCNA analysis of genes in the GSE16134 and GSE10334 datasets, we identified seven gene modules. Correlation analysis determined that the “ME turquoise” module was most positively associated with periodontitis (Figure 2C and Figure S1), suggesting its potential role in disease progression. Cross-analysis between the hub genes identified by WGCNA and the DEGs revealed 471 overlapping genes, which may play key roles in the onset and progression of periodontitis (Figure 2D). GO analysis showed that the intersecting genes were primarily involved in immune responses and leukocyte migration, including processes like lipopolysaccharide response, neutrophil migration, and B cell activation, and were associated with cellular structures related to immune cells such as the endoplasmic reticulum lumen, extracellular matrix, and NADPH oxidase complex. In terms of molecular function, these genes were involved in various activities including peptidase activity, cytokine and receptor binding, among others (Figure 2E,F). KEGG analysis indicated that these DEGs were enriched in several immune-related pathways, including pathogen infection, natural killer cell-mediated cytotoxicity, IL-17 signaling, and TNF signaling pathways. Additionally, these genes were closely associated with cell signaling and structural pathways such as cell adhesion molecules, chemokine signaling, and leukocyte transendothelial migration (Figure 2G).

### 3.2. Refinement of Core Gene Features Related to Periodontitis via Machine Learning

**Figure 3:**
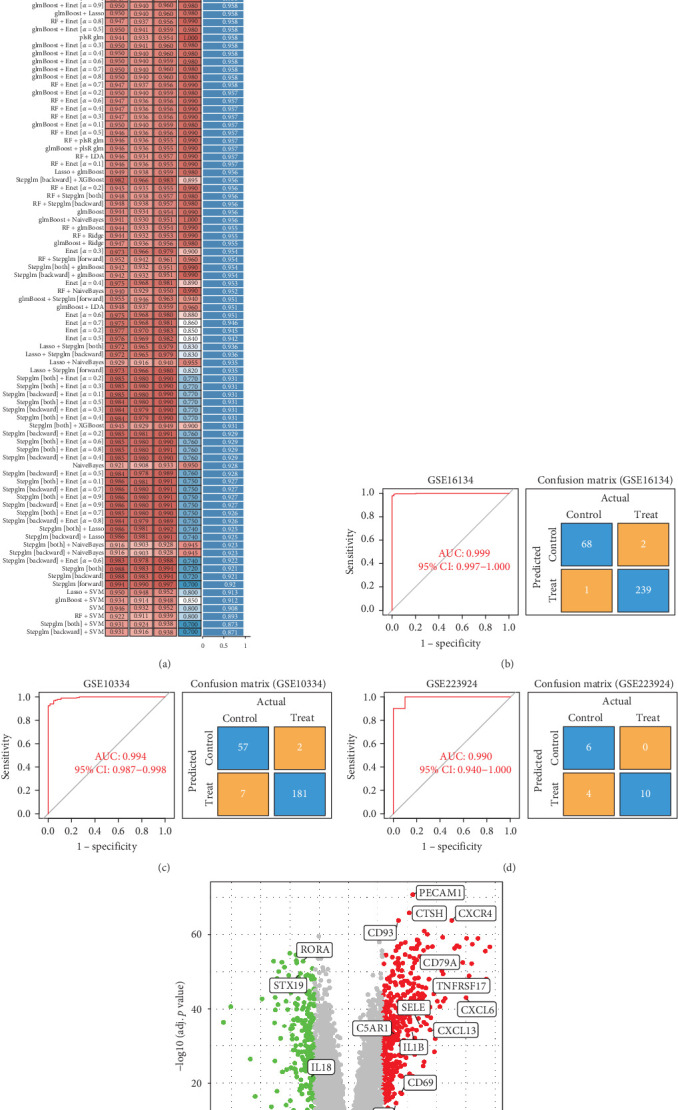
Refining the core gene characteristics associated with periodontitis through machine learning. (A) Performance comparison of prediction models based on different machine learning methods. (B–D) Receiver operating characteristic (ROC) curves and confusion matrices for the GSE16134, GSE10334, and GSE223924 datasets. (E) Volcano plot showing the differential analysis of the 19 candidate genes identified by XGBoost.

To identify candidate genes related to periodontitis, we cross-referenced the module genes obtained from WGCNA with the DEGs identified by limma analysis. The resulting periodontitis-related genes were then subjected to an integrated machine learning program to further refine core gene features significantly impacting periodontitis. Various algorithms were used for model training and validation, including elastic net, lasso regression, ridge regression, Stepglm, RF, GBM, SVM, DT, NB, KNNs, and XGBoost. Among the results, models incorporating XGBoost components exhibited the highest AUC values, indicating superior performance of the XGBoost model in the classification tasks for periodontitis (Figure 3A). The XGBoost model demonstrated excellent predictive ability in the GSE16134, GSE10334, and GSE223924 datasets (Figure 3B–D). Through the XGBoost model, 19 core genes were identified (Figure 3E).

### 3.3. MR Analysis Reveals Causal Relationships Between Periodontitis and Key Genes

**Figure 4:**
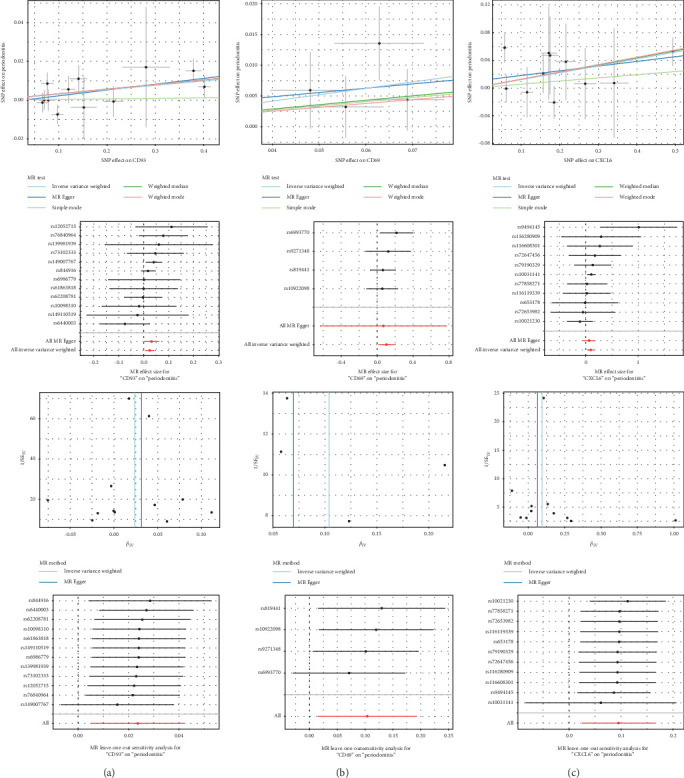
Mendelian randomization (MR) analysis revealing the causal relationships between periodontitis and key genes. (A–C) Scatter plots, forest plots, and funnel plots for CD93, CD69, and CXCL6, respectively, showing the causal effects on periodontitis risk, evaluating the causal effects of individual Single nucleotide polymorphisms (SNPs), and assessing overall heterogeneity. Leave-one-out analysis further visualizes the independent impact of each gene on periodontitis risk.

In this study, we conducted MR analysis using eQTL and pQTL data for 19 candidate genes to assess their causal relationships with periodontitis. The results showed that CD93, CD69, and CXCL6 were all significantly causally associated with the occurrence of periodontitis. The analysis for CD93 (OR = 1.024, 95% CI = 1.005–1.043, and *p*=0.0117), CD69 (OR = 1.110, 95% CI = 1.015–1.213, and *p*=0.0224), and CXCL6 (OR = 1.100, 95% CI = 1.025–1.180, and *p*=0.0080) did not show pleiotropic bias. Funnel plots and MR Egger regression validated the robustness and unbiased nature of the results. Sequential SNP removal analysis further supported the causal relationships between these genes and periodontitis (Figure 4A–C).

### 3.4. Molecular Characteristics of CD93, CD69, and CXCL6 in Periodontitis

**Figure 5:**
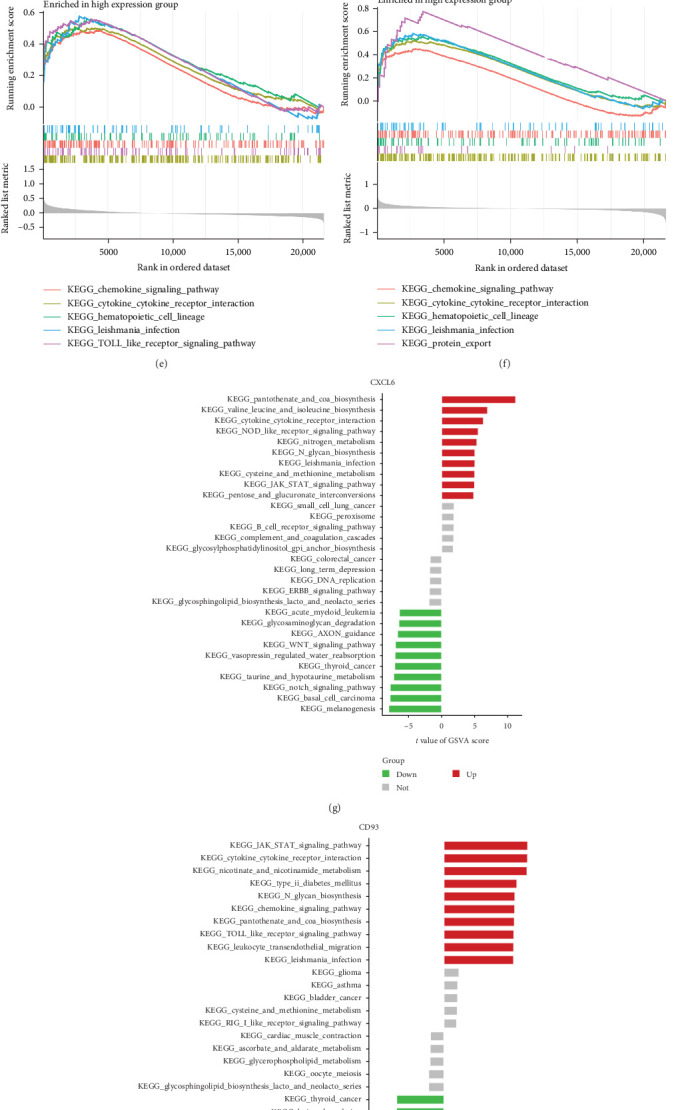
Molecular characteristics of CD93, CD69, and CXCL6 in periodontitis. (A) Expression analysis of CD93, CD69, and CXCL6 in normal and periodontitis tissues. (B) Correlation analysis of the three genes, CD93, CD69, and CXCL6. (C) Receiver operating characteristic (ROC) curves of CD93, CD69, and CXCL6 in the periodontitis datasets. (D–F) Gene set enrichment analysis (GSEA) analysis of CD93, CD69, and CXCL6 in the periodontitis datasets. (G–I) Gene set variation analysis (GSVA) analysis of CD93, CD69, and CXCL6 in the periodontitis datasets.

We conducted a detailed analysis of the expression levels of CD93, CD69, and CXCL6 in normal and periodontitis tissues, revealing that all three genes were significantly upregulated in periodontitis tissues (Figure 5A). Correlation analysis showed statistically significant correlations between CD69 and CD93, CD69 and CXCL6, and CD93 and CXCL6, with correlation coefficients of 0.16, 0.31, and 0.44, respectively (Figure 5B). In the evaluation of diagnostic potential, CD93′s ROC curve performed the best, with an AUC value of 0.901, indicating high diagnostic accuracy, followed by CXCL6 with an AUC of 0.843, and CD69 with an AUC of 0.735 (Figure 5C). To further explore the roles of these genes in the pathogenesis of periodontitis, we conducted GSEA and GSVA. GSEA results indicated that CD93, CD69, and CXCL6 were significantly enriched in several inflammation-related biological processes (Figure 5D–F), while GSVA analysis confirmed that these genes exhibited differential expression patterns under various inflammatory conditions (Figure 5G–I).

### 3.5. Immune Cell Infiltration and Inflammatory Marker Gene Analysis in Periodontitis

**Figure 6:**
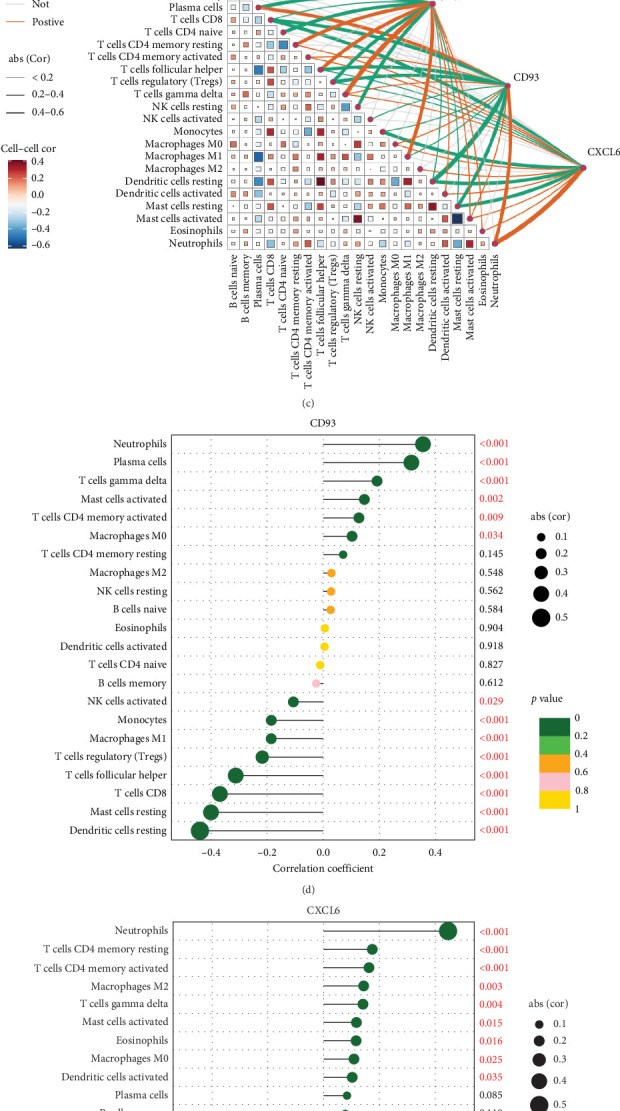
Analysis of immune cell infiltration and inflammatory marker genes in periodontitis. (A) Relative distribution of 22 immune cell types in normal and periodontitis samples. (B) Differential analysis of 22 immune cell types in normal and periodontitis samples. (C) Analysis of the relationships between CD93, CD69, CXCL6, and immune cells. (D) Analysis of immune cells associated with CD93 in periodontitis. (E) Analysis of immune cells associated with CD93 in periodontitis. (F) Analysis of immune cells associated with CXCL6 in periodontitis.

We conducted an in-depth analysis of immune cell infiltration differences between periodontitis patients and healthy controls, revealing significant differences in immune cell distribution between periodontitis and control samples (Figures 6A,B). We further explored the relationships between key inflammatory genes CD93, CD69, and CXCL6 and various immune cell types (Figure 6C). CD69, an early marker of immune cell activation, is mainly expressed in T cells, B cells, and natural killer cells. Our study found that CD69 was positively correlated with eosinophils and activated dendritic cells, suggesting that these cells may jointly upregulate CD69 during immune activation to participate in inflammatory responses. Conversely, CD69 was negatively correlated with regulatory T cells (Tregs), reflecting a potential opposition between immune cells with high CD69 expression and immunosuppressive cell functions (Figure 6D).

CD93, a glycoprotein expressed on various immune cells, was positively correlated with CD4 memory activated T cells, gamma-delta T cells, activated mast cells, plasma cells, and neutrophils, while showing negative correlations with activated NK cells, resting dendritic cells, resting mast cells, CD8 T cells, and Tregs (Figure 6E). CXCL6, a member of the CXC chemokine family, is mainly produced by macrophages and functions primarily in regulating immune cell migration, particularly neutrophils. The analysis revealed that CXCL6 was positively correlated with neutrophils, activated dendritic cells, M0 and M2 macrophages, eosinophils, activated mast cells, gamma-delta T cells, and CD4 memory activated and resting T cells, while showing negative correlations with Tregs, resting mast cells, CD8 T cells, resting dendritic cells, and monocytes (Figure 6F).

### 3.6. Single-Cell Analysis Reveals Cell-Specific Expression of Periodontitis-Related Genes

**Figure 7:**
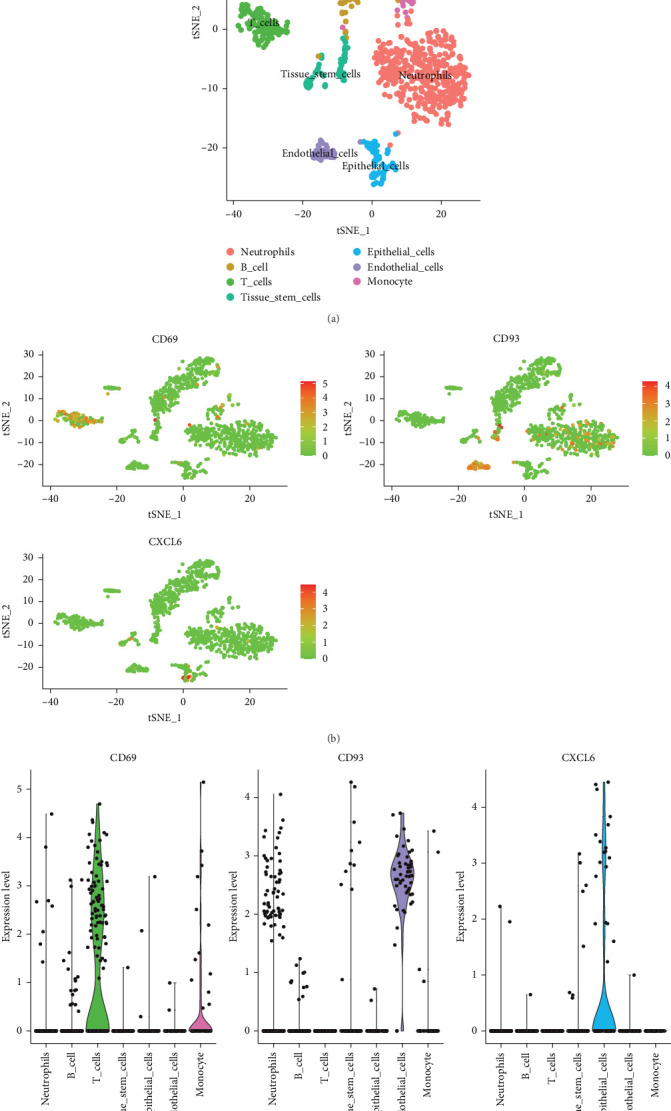
Single-cell analysis revealing the cell-specific expression of periodontitis-related genes. (A) UMAP plot of single cells from the GSE171213 cohort of periodontitis. (B) Relative expression of CD93, CD69, and CXCL6 genes across different cell types. (C) Violin plot showing the expression of CD93, CD69, and CXCL6 genes in different cell types.

To explore the connections between CD93, CD69, and CXCL6 and periodontitis, we performed single-cell data analysis. Seven cell types were identified in the GSE171213 dataset, including neutrophils, B cells, T cells, tissue stem cells, epithelial cells, endothelial cells, and monocytes (Figure 7A). Interestingly, CD93, CD69, and CXCL6 exhibited differential distribution across different cell types (Figure 7B). CD69 was primarily expressed in T cells and monocytes, CD93 was mainly expressed in endothelial cells, and CXCL6 was predominantly expressed in epithelial cells (Figure 7C).

## 4. Discussion

Periodontitis is a widespread chronic inflammatory disease that affects approximately 45%–50% of adults worldwide [1, 2], with severe periodontitis impacting around 11.2% of the population [3]. In addition to causing serious oral health issues, periodontitis is closely linked to systemic health problems such as cardiovascular disease, diabetes, and preterm birth, posing significant public health challenges [40–43]. Traditional research has primarily relied on clinical and pathological indicators to diagnose and assess the severity of the disease [10, 44]. However, these methods are limited in their ability to reveal the underlying molecular mechanisms of the disease. In recent years, the application of machine learning technology in periodontitis research has brought new breakthroughs in disease diagnosis and mechanism exploration [14, 45]. By analyzing large-scale biomedical data, particularly through machine learning models, it is possible to identify potential disease biomarkers and pathological processes, thereby, improving disease prediction accuracy and advancing personalized treatment development [12]. In this study, we innovatively combined machine learning with MR to explore molecular biomarkers and pathological mechanisms of periodontitis.

Using transcriptomic data from the GEO database [46], we identified 682 significantly DEGs between periodontitis and normal samples, including 447 upregulated and 235 downregulated genes. Through WGCNA, we identified seven gene modules closely related to periodontitis and, through cross-analysis, identified 471 core candidate genes. Further GO and KEGG pathway analyses revealed that these genes are primarily involved in critical biological processes such as immune responses, leukocyte migration, and bacterial responses. They play important roles in immune reactions such as neutrophil migration and B cell activation and are involved in inflammation-related pathways such as the IL-17 signaling pathway and the TNF signaling pathway. such as the IL-17 and TNF signaling pathways [47, 48], highlights their potential role in exacerbating the inflammatory environment in periodontitis. The IL-17 signaling pathway is known to promote the recruitment of neutrophils and other immune cells to the site of infection, thereby amplifying the inflammatory response. Similarly, the TNF signaling pathway is a key regulator of inflammation and has been implicated in the destruction of periodontal tissues through the promotion of osteoclastogenesis and tissue breakdown. The activation of these pathways in periodontitis suggests that the identified DEGs may contribute to the progression of the disease by sustaining chronic inflammation and driving tissue damage.

By integrating 113 machine learning models, we found that the XGBoost algorithm was most effective in identifying 19 core genes associated with periodontitis. MR analysis further investigated the causal relationships between these genes and periodontitis. MR, which leverages genetic variation as instrumental variables, helps overcome confounding factors and reverse causality issues inherent in traditional observational studies, providing more robust causal inferences. Our MR analysis, using eQTL and pQTL data, identified significant causal relationships between periodontitis and the genes CD93, CD69, and CXCL6.

CD93 is critical in regulating immune cell adhesion and migration, key processes in the inflammatory response of periodontitis [49]. Beyond its well-documented roles, CD93 also influences the maturation and differentiation of immune cells, potentially modulating the immune landscape in periodontal disease. CD69, an early activation marker, plays a role in initiating and regulating immune responses, potentially exacerbating the inflammatory environment in periodontal tissues [50]. It also facilitates lymphocyte retention in inflamed tissues, enhancing local immune response and its dysregulation could contribute to chronic inflammation characteristic of periodontitis. CXCL6, essential for neutrophil chemotaxis [51], highlights the dual role of neutrophils in both controlling bacterial infection and contributing to tissue destruction in periodontitis. Additionally, CXCL6 mediates interactions between neutrophils and endothelial cells, crucial for the recruitment and transmigration of these immune cells during the immune response.

Immune cell infiltration analysis demonstrated that these genes are highly expressed in periodontal tissues and are closely associated with immune cell infiltration. This suggests that CD93, CD69, and CXCL6 are not only involved in the immune responses driving periodontitis but may also serve as promising therapeutic targets. Additionally, to further explore the specific roles of these key genes in the pathological process of periodontitis, we analyzed the cell-type-specific expression patterns of CD93, CD69, and CXCL6 using single-cell RNA sequencing. The results showed that CD69 is primarily expressed in T cells and monocytes, indicating its role in immune regulation and cell activation [52, 53]; CD93 is mainly expressed in endothelial cells, supporting its function in angiogenesis and immune cell adhesion [54]; while CXCL6 is predominantly expressed in epithelial cells, suggesting its importance in tissue inflammatory responses and chemokine-mediated immune cell recruitment [55]. GSVA and GSEA analyses further confirmed the significant enrichment of these genes in inflammation-related pathways, indicating their critical roles in the immune response and pathological progression of periodontitis. The single-cell analysis elaborated the cellular localization and functional involvement of CD93, CD69, and CXCL6 within specific immune cell types, enriching the mechanistic insights provided by MR. This dual approach not only confirms the genetic predisposition to periodontitis but also provides a cellular basis for these associations, demonstrating how these genes contribute to the complex immune landscape of periodontitis. By validating the causal inferences drawn from MR with precise cellular expression patterns, this study underscores the potential of integrating genetic and transcriptomic data to delineate disease pathophysiology.

In summary, our study provides new insights into the molecular mechanisms of periodontitis and offers potential targets for the development of personalized treatment strategies. The combination of MR analysis and single-cell sequencing provides valuable tools for a deeper understanding of the causal relationships between periodontitis and specific genes, as well as their specific roles in different cell types. Our findings enhance personalized medicine strategies for periodontitis by identifying key biomarkers CD93, CD69, and CXCL6 and their links to the disease through MR. This could lead to treatments tailored to genetic profiles, particularly for patients with high expression levels of these genes, who might benefit from targeted therapies. Additionally, employing machine learning models to predict disease progression or treatment responses could enable more precise, effective, and patient-specific management strategies. However, despite the strong data support, experimental validation of these genes' specific roles in periodontitis remains necessary. Future research should focus on the dynamic expression and functional studies of these genes during disease progression to further deepen our understanding of the complex pathophysiology of periodontitis and facilitate the translation to clinical applications [56]. In addition, our dataset lacks broad ethnic diversity, which may affect the generalizability of our findings across different populations. Future studies should aim to validate our identified biomarkers in larger and more ethnically diverse cohorts. The functional roles of these biomarkers in periodontitis have yet to be fully elucidated. Conducting functional validation studies, both in vitro and in vivo, could clarify the mechanisms through which these biomarkers influence the pathogenesis of periodontitis, potentially leading to novel therapeutic targets.

## 5. Conclusion

This study comprehensively revealed the molecular mechanisms of periodontitis by combining machine learning, MR analysis, and single-cell sequencing technology, successfully identifying key immune-related biomarkers. The results showed significant causal relationships between CD93, CD69, and CXCL6 and periodontitis. Additionally, we discovered that these genes have specific expression patterns in different immune cells, highlighting their important roles in the pathological processes of periodontitis. These findings not only provide new insights into the molecular mechanisms of periodontitis but also offer potential targets for the development of early diagnostic and personalized treatment strategies.

## Data Availability

The datasets used in this study are available in the Gene Expression Omnibus (GEO) database under the accession numbers “GSE16134,” “GSE10334,” “GSE223924,” and “GSE171213.” The expression quantitative trait loci (eQTL) data used for Mendelian randomization analysis can be accessed from the IEU Open GWAS database (https://gwas.mrcieu.ac.uk/), and the protein quantitative trait loci (pQTL) data can be accessed from the deCODE Genetics database (https://www.decode.com/summarydata/).

## Supporting Information

Supporting Information In this study, we utilized weighted gene co-expression network analysis (WGCNA) to identify key modules and genes associated with immune-related biomarkers in periodontitis. The detailed screening process and results are provided in the Supporting Information. Figure S1. (A) Scale Independence. (B) Merged Dynamic.
