# Supplementary figures and images for: Machine Learning and Mendelian Randomization Reveal Molecular Mechanisms and Causal Relationships of Immune-Related Biomarkers in Periodontitis
Source: Mediators Inflamm. 2024 Dec 16;2024:9983323. doi: 10.1155/mi/9983323 (PMC11666315; doi:10.1155/mi/9983323)

**A**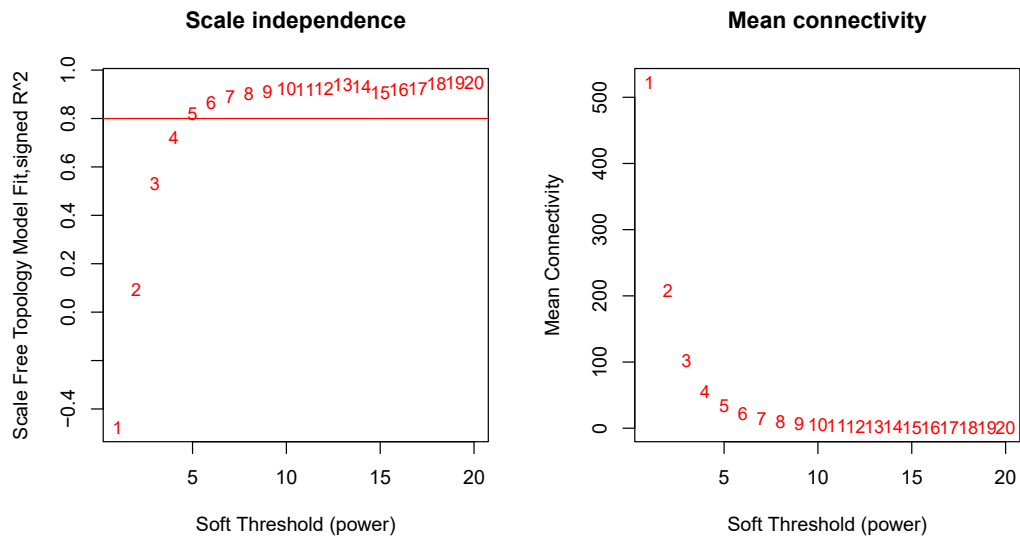**B**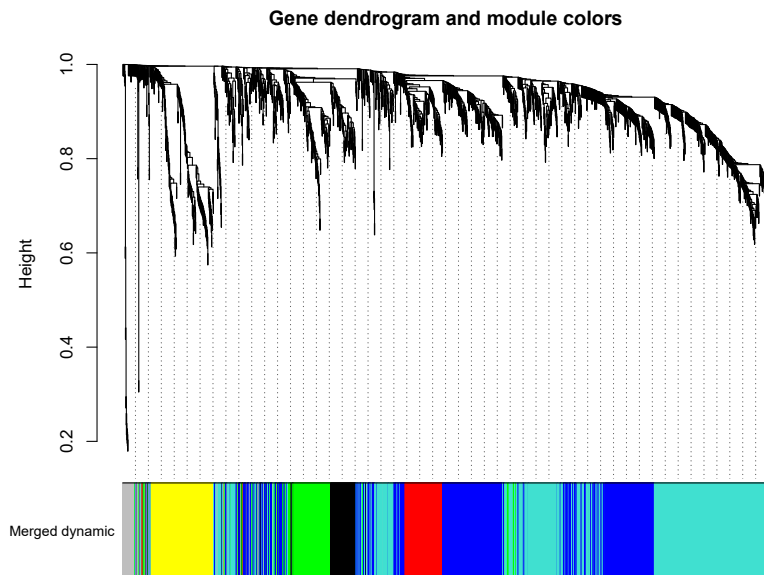

Supplement: Supporting Information — In this study, we utilized weighted gene co-expression network analysis (WGCNA) to identify key modules and genes associated with immune-related biomarkers in periodontitis. The detailed screening process and results are provided in the Supporting Information. Figure S1. (A) Scale Independence. (B) Merged Dynamic. [file 9983323.f1.pdf]
